# Prognostic factors of radiation dermatitis following passive-scattering proton therapy for breast cancer

**DOI:** 10.1186/s13014-018-1004-3

**Published:** 2018-04-19

**Authors:** Xiaoying Liang, Julie A. Bradley, Dandan Zheng, Michael Rutenberg, Daniel Yeung, Nancy Mendenhall, Zuofeng Li

**Affiliations:** 1 Department of Radiation Oncology, University of Florida, Gainesville, FL USA; 2 Department of Radiation Oncology, University of Nebraska Medical Center, Omaha, NE USA

**Keywords:** Radiation dermatitis, Breast cancer, Passive-scattering proton therapy, Logistic regression analysis

## Abstract

**Background:**

To identify prognostic factors for grade 3 radiation dermatitis following passive-scattering proton therapy for breast cancer.

**Methods:**

This retrospective study included data on 23 (11 post-mastectomy and 12 post-lumpectomy) breast cancer patients who underwent proton therapy with the passive scattering technique in our institute from 2012 to 2016. Each patient received 50–50.4 cobalt Gy equivalent (CGE) at 1.8 or 2 CGE per daily fraction. Logistic regression analysis was performed to identify prognostic factors for grade 3 skin toxicity. Receiver operating characteristic (ROC) curve analysis and the area under the curve (AUC) were used to evaluate the performance of the models.

**Results:**

43% of the studied patients developed grade 3 radiation dermatitis. The dose-volume histogram (DVH) parameters of V52.5CGE and D10cm^3^ to skin5mm were correlated with grade 3 radiation dermatitis in both univariate and multivariate logistic regression analyses. Univariate logistic regression analysis suggested that D10cm^3^ to skin5mm (AUC = 0.69) and V52.5CGE to skin5mm (AUC = 0.70) were prognostic for grade 3 skin toxicity. The models using the combination of D10cm^3^ to skin5mm or V52.5CGE to skin5mm with breast volume marginally increased the AUC to 0.72 and 0.73, respectively. Models using the combination of D10cm^3^ to skin5mm or V52.5CGE to skin5mm with history of smoking increased the AUC to 0.75 and 0.83, respectively.

**Conclusion:**

In the current study, we identified prognostic factors for grade 3 radiation dermatitis in patients treated with passive-scattering proton therapy for breast cancer. This study provides promising tool for identifying high risk patients for whom treatment plan adjustment could be done to reduce the risk of radiation-induced grade 3 skin toxicity.

## Background

Post-lumpectomy or post-mastectomy irradiation is known to reduce local recurrence and increase overall survival compared with surgery alone [1, 2]. Despite survival gains, radiation is associated with an increased risk of heart and lung disease (REF) [3, 4]. Compared with conventional photon-based radiation therapy, proton therapy can deliver radiation therapy to breast cancer targets with minimal dose to the heart and lung [5–7]. However, patients can experience increased radiation dermatitis with passive-scattering proton therapy compared with photon therapy [8]. Due to the nature of passive-scattering proton beams and the proximity of the target volume to skin in breast cancer, the skin may receive the full prescription dose or higher.

Radiation dermatitis is the predominant acute side effect of radiotherapy for breast cancer. Acute reactions can impact quality of life and compromise the delivery of cancer treatment. Radiation dermatitis can progress from erythema to dry desquamation to moist desquamation and even to ulceration [9], after which permanent skin changes may develop that affect cosmesis. Severe acute reactions can lead to interruption or premature discontinuation of radiation therapy and potentially negatively influencing cancer control and prognosis. The majority of measures currently available to prevent these acute reactions are proper skin hygiene, hydration, topical plant based agents such as aloe and calendula, topical steroids and mepitel film. Numerous trials aiming to prevent acute radiation adverse effects to skin using creams, hygiene or semi-permeable dressing have been reported, but their efficacy in prevention of skin reactions has not been consistently demonstrated across studies [10–13].

The purpose of this study is to identify prognostic factors of developing grade 3 radiation dermatitis following passive-scattering proton therapy, so that for those patients who are at high risk of severe skin toxicity, the treatment plans can be adjusted to reduce the risk.

## Method

### Patient population and end point

In this study, we retrospectively evaluated 23 (11 post-mastectomy and 12 post-lumpectomy) patients with breast cancer who were treated with passive-scattering proton technique in our institute from 2012 to 2016. The study was approved by the institutional review board. All patients were simulated in the supine position with arms above their heads using a wingboard and Vac-Lok bag on a Philips Brilliance Big Bore CT (Philips Healthcare, Andover, MA). Treatment was planned using Varian Eclipse TPS (V13.5) (Varian Medical Systems, Palo Alto, CA) with passive-scattering proton therapy. Two en face angles between 0^o^ and 60^o^ were used to minimize the effect of respiratory breathing and to improve the homogeneity of dose distribution [5, 14]. Each patient received 50–50.4 cobalt Gy equivalent (CGE) at 1.8 or 2 CGE per daily fraction (5 and 18 patients, respectively). For those patients who were treated with 1.8 CGE per fraction, an α/β of 10 CGE for acute skin reaction was used to convert the dose to 2 CGE per fraction equivalent dose using Eq. 1 [15]:

1 $$ {EQD}_2=D\left(1+\frac{d}{\alpha /\beta}\right)/\left(1+\frac{2}{\alpha /\beta}\right) $$

Patients’ dermatitis reactions were evaluated based on CTCAE v4.0 in a prospective fashion at weekly on-treatment visits and at every follow-up. All patients experienced at least moderate (grade 2) skin reactions consisting of brisk erythema or patchy moist desquamation confined to skin folds; 43% (10/23) developed grade 3 radiation dermatitis, characterized by moist desquamation in areas other than skin folds and creases, bleeding induced by minor trauma or abrasion. The primary endpoint for this study was grade 3 skin toxicity.

#### Studied parameters

The study parameters were selected based on existing literatures on skin toxicity from breast radiotherapy [16–20] as well as our clinical practice:

- Patient-specific and tumor-specific factors: breast volume (cm^3^) [16–18], and planning target volume (cm^3^) [19], body mass index (BMI) [20], age [20], history of smoking (never smoker vs. any smoking history) [17, 20], and existence of diabetes. Planning target is a combined and smoothed structure comprised of the breast PTV and nodal PTV.
- Dose-volume histogram (DVH) parameters for “skin5mm”, a layer structure of 5 mm inward from the body contour; volumes (cm^3^) received 25 CGE, 47.5 CGE, 50 CGE, and 52.5 CGE (V25CGE, V47.5CGE, V50CGE, and V52.5CGE, respectively) and doses delivered to 150 cm^3^, 100 cm^3^, 30 cm^3^, and 10 cm^3^ (D150cm^3^, D100cm^3^, D30cm^3^, and D10cm^3^, respectively) of the skin5mm [21].

#### Data analysis

The Mann-Whitney U-test was used on the continuous variables and the Pearson’s chi-squared test was used on the categorical variables, to test for differences between patients who did and did not develop grade 3 radiation dermatitis. A value of *p* ≤ 0.05 was considered significant. Logistic regression analyses [22, 23] were performed to identify prognostic factors of grade 3 skin toxicity. Collinearity of the covariates can distort the interpretation of a multivariate regression model [24–26]. Therefore, correlations among the parameters were studied. Only those non-correlated parameters (with *r* < 0.3) were used in the multivariate logistic regression analysis. To avoid over fitting, we tested the performance of our model by means of leave-one-out. The leave-one-out cross validation (LOOCV) refers to the process of removing 1 patient, used as the validation sample, and reconstructing the model using the reduced sample set. The new model is then tested for predictive accuracy against the excluded patient; the process is repeated 23 times (each time with a different excluded patient).

The receiver operating characteristic (ROC) curve was plotted using the validation results from LOOCV and the area under the curve (AUC) [27] was used to evaluate the performance of the models in prediction of grade 3 radiation dermatitis. An ROC curve is a plot of the true positive fraction (sensitivity) versus the false positive fraction (1-specificity). A value of AUC = 1 is a perfect prediction, while a value of 0.5 is equivalent to a random guess. For those with AUC > 0.65, a reconstruction of the models on all 23 patients was performed and the coefficients of the model were presented.

## Results

**Fig. 1:**
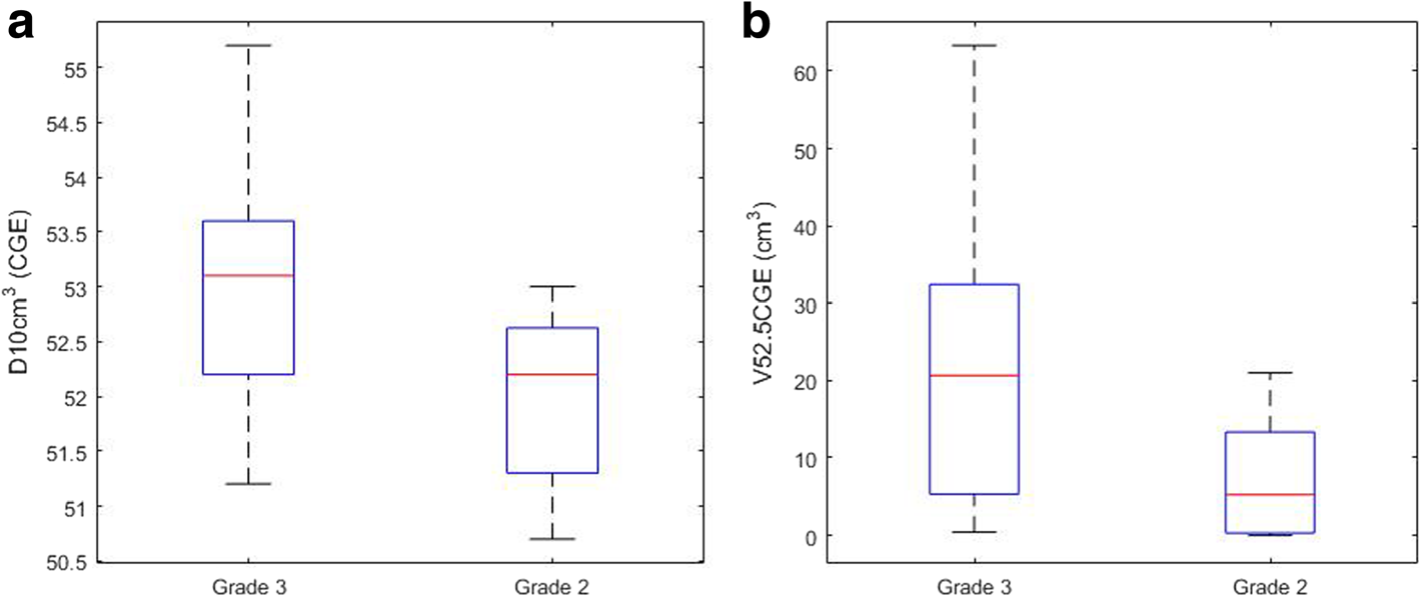
Box plot for (**a**) D10cm^3^ to skin5mm and (**b**) V52.5CGE to skin5mm of grade 2 and grade 3 radiation dermatitis

Table 1 show the median (range) and *p* values of the Mann-Whitney U-test on the continuous variables. Statistically significant differences were observed for D10cm^3^ and V52.5CGE to skin5mm. Figure 1a and b show the data distribution for D10cm^3^ and V52.5CGE to skin5mm, respectively. Patients with grade 3 radiation dermatitis received higher D10cm^3^ to skin5mm and had larger volumes of skin5mm that received 52.5 CGE. The results of the Pearson’s chi-squared test on the categorical variables are also shown in Table 1. Statistically significant differences were observed for history of smoking.

**Table 1:** Median (range) and *p* values of the Mann-Whitney U-test on continuous variables, and percentage and *p* values of the Pearson’s chi-squared test on categorical variable, in groups with grade 2 and grade 3 skin toxicity

	Grade 3	Grade 2	
Continuous variables	Median (range)	Median (range)	Mann-Whitney U-test *p*-values
V25CGE (cm3)	257.8 (194.8–330.6)	260.8 (197.7–355.9)	0.93
D150cm3 (CGE)	46.6 (40–48.9)	46.7 (40.4–49.1)	0.69
D100cm3 (CGE)	49.5 (46.4–50.7)	48.9 (46.8–50.3)	0.19
D30cm3 (CGE)	52.1 (49.3–53.9)	51.5 (50.0–52.1)	0.07
D10cm3 (CGE)	53.1 (51.2–55.2)	52.2 (50.7–53.0)	**0.03**
V50CGE (cm3)	83.0 (22.7–119.8)	68.3 (20.1–114.7)	0.28
V47.5CGE (cm3)	140.3 (76.9–173.8)	118.5 (91.8–168.6)	0.13
V52.5CGE (cm3)	20.7 (0.4–63.3)	5.2 (0–21.0)	**0.03**
breast volume (cm3)	796.5 (287.2–1746.1)	928.8 (221.7–1328.9)	0.31
planning target volume (cm3)	1224.3 (644.8–2394.0)	1166.0 (490.5–1760.7)	0.64
body mass index	28.7 (23.8–52.1)	29.4 (21.9–47.2)	0.83
age (years)	64 (10)	60 (11)	0.66
Categorical variables	Percentage	Percentage	Pearson’s chi-squared test *p*-values
history of smoking	80.0%	30.8%	**0.02**
existence of diabetes	30.0%	23.1%	0.71

Statistical significance is indicated in bold

*Abbreviations***:** *CGE* cobalt gray equivalent

Univariate logistic regression analysis was performed on each parameter. The AUC values from LOOCV were used to evaluate the model performance in predicting grade 3 radiation dermatitis (Table 2). Using an AUC = 0.65 as the cutoff value, univariate logistic regression analysis suggested that D10cm^3^ to skin5mm and V52.5CGE to skin5mm with AUC of 0.69 and 0.70, respectively, were prognostic for grade 3 skin toxicity. Figure 3a and b shows the ROC curves on the D10cm^3^ model and the V52.5CGE model, respectively. Although history of smoking shows statistically significant differences (*p* = 0.02), the AUC value of 0.55 suggests a poor prognostic power by this single parameter.

**Table 2:** AUC values from LOOCV in predicting grade 3 radiation dermatitis

Metric	Parameter	AUC	95% CI	
Single parameter	(a) D10cm^3^	0.69	0.51	0.92
	(b) V52.5CGE	0.70	0.51	0.92
Two-parameter metric 1(c)	breast volume + D10cm^3^	0.72	0.52	0.93
Two-parameter metric 2 (d)	breast volume + V52.5CGE	0.73	0.51	0.94
Two-parameter metric 3 (e)	history of smoking+D10cm^3^	0.75	0.57	0.94
Two-parameter metric 4 (f)	history of smoking+V52.5CGE	0.83	0.63	0.96

The dose and volume parameters are related to skin5mm

*Abbreviations*: *AUC* area under the curve, *CGE* cobalt gray equivalent, *CI* confidence interval

**Fig. 2:**
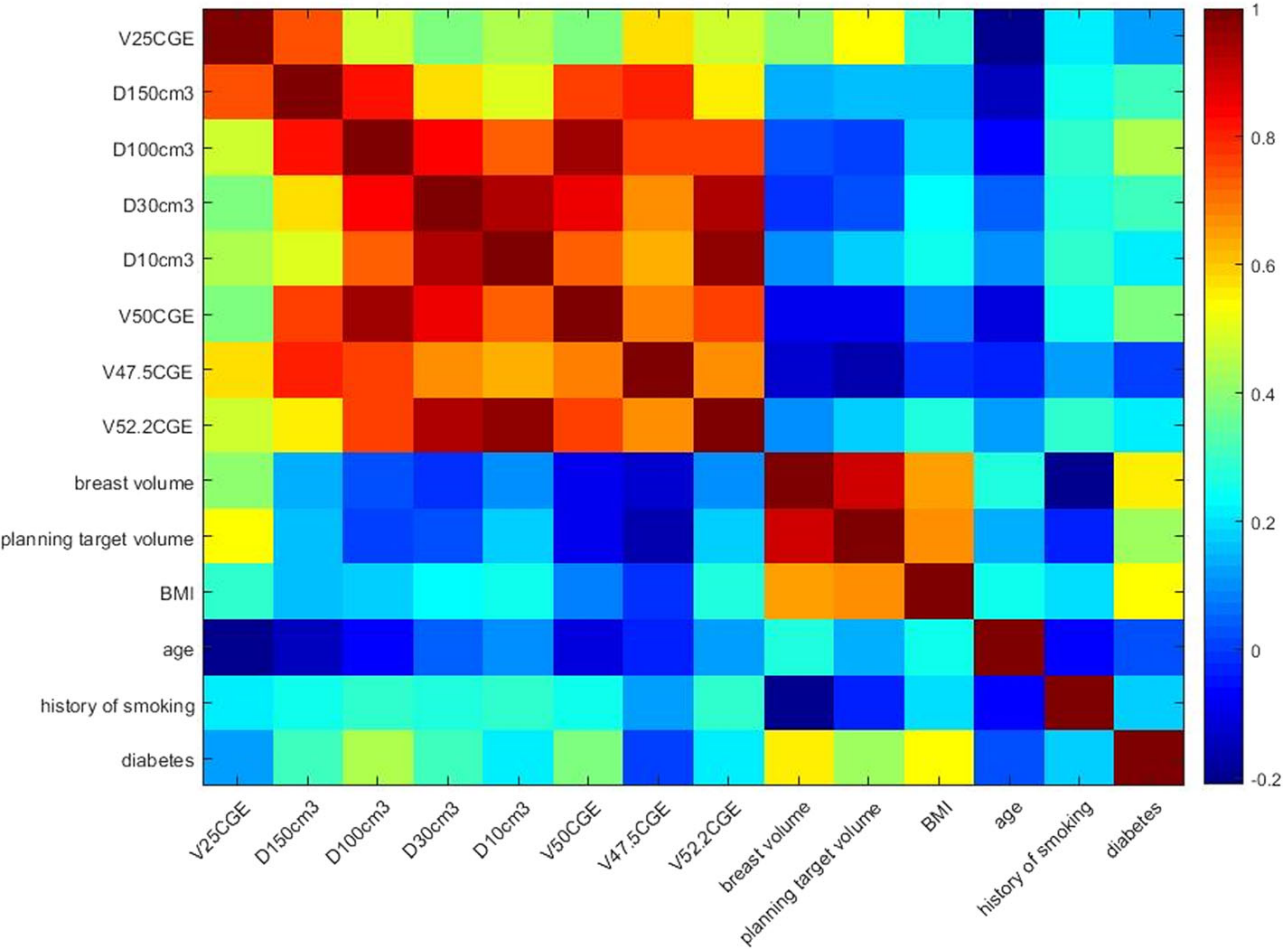
Correlation of the studied parameters. The strongest correlation (*r* = 1) is indicated in red, and no correlation (*r* = 0) is indicated in blue

**Fig. 3:**
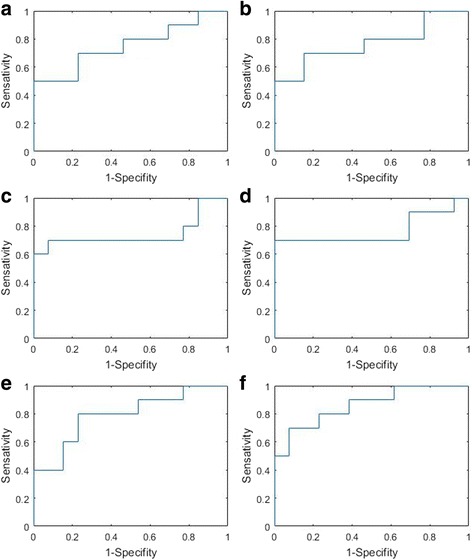
Receiver operating characteristic curves from the LOOCV validation results for the **a**) D10cm^3^ model, **b**) V52.5CGE model, **c**) breast volume + D10cm^3^ model, **d**) breast volume + V52.5CGE model, **e**) history of smoking + D10cm^3^ model, and **f**) history of smoking + V52.5CGE model, in prediction of grade 3 radiation dermatitis

The correlations among the parameters were studied and illustrated in a color map in Fig. 2, where the strongest correlation (*r* = 1) is indicated in red, and no correlation (*r* = 0) is indicated in blue. Only those non-correlated parameters (with *r* < 0.3) were used in the multivariate logistic regression analysis. The AUC values from the results of LOOCV of multi-parameter models are shown in Table 2. Those models with improved AUC values compared with single parameter model are shown. The two-parameter models of breast volume + D10cm^3^ and breast volume + V52.5CGE increased the AUC marginally from 0.69 and 0.70 for the one parameter model to 0.72 and 0.73, respectively. The models of history of smoking + D10cm^3^ and history of smoking + V52.5CGE increased the AUC to 0.75 and 0.83, respectively. The correlation between breast volume and the D10cm^3^ to skin5mm or the V52.5CGE to skin5mm were very low, with r values of 0.09 and 0.10 and *p* values of 0.67 and 0.65, respectively. The correlation between history of smoking and the D10cm^3^ to skin5mm or the V52.5CGE to skin5mm were low, both with r value of 0.29 and p value of 0.18. Figure 3 shows the LOOCV ROC curves on the (c) breast volume + D10cm^3^ model, (d) breast volume + V52.5CGE model, (e) history of smoking + D10cm^3^ model, (f) history of smoking + V52.5CGE model in prediction of grade 3 radiation dermatitis. For those with AUC > 0.65, a reconstruction of the models on all 23 patients were performed and the logistic regression coefficients and *p* values of these models are shown in Table 3.

**Table 3:** The logistic regression coefficients and *p* values

Metric	Parameter	Coefficient	95% CI		*P*-value
Single parameter	(a) D10cm^3^	1.26	−0.01	2.53	0.05
	constant	−0.32	−1.25	0.62	
	(b) V52.5CGE	1.46	0.04	2.88	**0.04**
	constant	−0.19	− 1.16	0.78	
Two-parameter metric 1 (c)	breast volume	−0.88	−2.00	0.24	0.14
	D10cm^3^	1.53	0.17	2.89	**0.03**
	constant	−0.40	−1.41	0.60	
Two-parameter metric 2 (d)	breast volume	−0.80	−1.97	0.37	0.16
	V52.5 CGE	1.65	0.26	3.04	**0.02**
	constant	−0.31	−1.35	0.74	
Two-parameter metric 3 (e)	history of smoking	1.12	0	2.24	**0.05**
	D10cm^3^	1.14	−0.07	2.35	0.06
	constant	−0.52	−1.65	0.60	
Two-parameter metric 4 (f)	history of smoking	1.18	0	2.35	**0.05**
	V52.5 CGE	1.27	−0.10	2.64	0.07
	constant	−0.48	−1.65	0.70	

Significant *p*-values are indicated in bold. The dose and volume parameters are related to skin5mm

*Abbreviations*: *CGE* cobalt gray equivalent, *CI* confidence interval

## Discussion

Studies have shown that proton therapy in the treatment of breast cancer reduces inadvertent radiation dose to the heart and lung, increase the therapeutic ratio of radiation therapy [5–7, 28]. However, the risk of a potential increase in skin toxicity [8] has long been considered a potential limiting factor in the clinical use of protons for breast cancer. The risk of radiation dermatitis is highly variable depending on the radiation target (breast vs. chest wall) as well as multiple patient and treatment factors. Previous studies [16–20, 29–35] conducted in the setting of photon therapy have attempted to identify potential factors associated with increased radiotherapy-induced skin toxicity. To the best of our knowledge, so far there are no similar publications on proton therapy.

The study by Parekh et al. [32] demonstrated that the risk of moist desquamation is much higher in the setting of post-mastectomy radiation therapy compared with the setting of breast conservation. In the present study, 4/12 the post-lumpectomy patients and 6/11 of the post-mastectomy patients developed grade 3 radiation dermatitis. Although no statistical conclusion could be drawn in our cohort due to the limited sample size, the same trend was observed as with the photon data. Treatment technique and dosimetric factor studies [19, 33–35] showed that a heterogeneity dose distribution and hotspot in PTV results in significantly more acute skin toxicity. The dosimetric parameters that used in those studies are on PTV rather than on skin itself. This might due to the skin sparing nature with photon therapy, the skin in general receives relatively low dose in photon therapy. Dose volume information on skin was use in the study of Pastore et al. [29], where a two-variable model including the skin receiving ≥30Gy and psoriasis is found to be predictive for acute radiation induced skin toxicity. In proton therapy, especially with passive scattering technique, the skin receives full prescription dose (~ 50 Gy). The changes of dose distributions over skin with proton therapy compared with photon therapy are more likely to affect the applicability of the findings from those literatures to the setting of proton therapy. In our study, higher D10cm^3^ and V52.5CGE to skin are associated with grade 3 acute radiation dermatitis. Various research groups found that breast volume [16–18], and smoking [20] are correlated with skin toxicity. In our study, we also found the breast volume and history of smoking played a role in the multi-parameter models. The breast volume + D10cm^3^ or V52.5CGE models indicate that when D10cm^3^ and V52.5CGE to skin5mm remain the same, women with a small breast volume have a higher likelihood of grade 3 radiation dermatitis. Although De Langhe et al. [17] and Kraus-Tiefenbacher et al. [20] found that larger breast volume was associated with increased risk of acute skin toxicity; neither investigated the effect of a combination of breast volume and dosimetric factors. A possible explanation is larger breast volumes have greater dose inhomogeneity compared to smaller breast volumes. The dose inhomogeneity may lead to partial hot spots followed by increased skin toxicity [36]. In our study, the coefficients of 1.65 and 1.53 and the *p* values of 0.02 and 0.03 on V52.5CGE and D10cm^3^ to skin5mm in the V52.5CGE or D10cm^3^ + breast volume models demonstrated the importance of hot spot influence on acute skin toxicity. The history of smoking + D10cm^3^ or V52.5CGE models indicate that patients with history of smoking have a higher likelihood of grade 3 radiation dermatitis. Age did not seem to play a role in any of our models, which is in agreement with Kraus-Tiefenbacher’s study [20]. BMI and existence of diabetes also seem not play a role in the current study.

This study is conducted on patients treated with passive-scattering proton technique. Proton therapy facilities have started to treat breast patients with the more advanced intensity modulated proton therapy (IMPT) [37, 38] that is enabled by pencil beam scanning (PBS) technique. In IMPT, as skin dose constraints can be added to the cost function during optimization, a reduction of skin dose is expected [39]. Due to the reduced skin dose afforded by PBS, lower incidence of severe skin toxicity could be expected than shown in our cohort of patients treated with passive-scattering proton technique. On the other hand, due to the substantial cost associated with starting or upgrading a proton facility, passive-scattering technique is still clinically used for breast cancer proton therapy at many facilities, where the findings of our study could be directly applicable. Moreover, even for PBS, the results from our study could potentially provide useful information such as on chest wall or inflammatory breast cancer treatment, where a full or near-full prescription skin dose is typically delivered. Needless to say, clinical validation study will be needed for such generalization. For post-lumpectomy patients, however, the different dose distributions with PBS compared with passive-scattering are more likely to affect the direct applicability of our results, although it remains to be investigated whether our findings may serve as upper limits of the relevant dosimetric parameters. We intend to perform additional studies in the future, on model refining and clinical validations on more diverse cohorts, once more patients are available for study.

Admittedly, this study had limited number of study subjects, which affected the robustness of our models. In addition, the individual reaction of the skin to radiation is complex and may be impacted by numerous factors that may be difficult to characterize and quantify. In this regard, it is important to note that this study is a hypothesis generating study to identify parameters that are prognostic for severe radiation dermatitis for breast cancer patients treated with passive-scattering proton technique rather than to generate a model. It should be clearly emphasized that we don’t intent to construct a robust prognostic model ready to be used as a clinical decision making criteria, but to use provide useful metrics for treatment planning to minimize severe radiation dermatitis. Nevertheless, further investigation and clinical validation are warranted. This will be done when more patient data become available for analysis/validation.

In the current study, history of smoking, breast volume and skin dose-volume histogram are proposed as a means to be prognostic for grade 3 skin toxicity, which is of key clinical relevance in proton therapy for breast cancer. Both patient-specific clinical parameters and treatment plan dosimetric factors have been assessed. The treatment plan can be adjusted if necessary to reduce the risk of severe radiation dermatitis. Given that the interest in proton therapy for the treatment of breast cancer has substantially increased over recent years and a large randomized trial (RADCOMP) of proton vs photon therapy for breast cancer patients just opened recently [40], the current study is deemed necessary even with the limitations discussed above.

## Conclusion

This study provides the first report of identifying prognostic factors for passive scattering proton therapy grade 3 radiation dermatitis in the treatment of breast cancer. The ROC curve analysis and AUC values from LOOCV showed that the DVH parameters of D10cm^3^ and V52.5CGE to skin5mm are correlated with grade 3 skin toxicity. More validation is required and further investigation is warranted.

## Abbreviations

AUC: area under the curve
BMI: body mass index
CGE: cobalt gray equivalent
ROC: receiver operating characteristic curve
